# Dilemma of Tocilizumab therapy for a patient with critical COVID‐19 disease and neutropenia: Case report and review of the literature

**DOI:** 10.1002/ccr3.5932

**Published:** 2022-05-27

**Authors:** Ahmad Al Bishawi, Shiema Abdalla, Marwa Askar, Wael Kanjo, Amal Sameer, Gihan Mustafa, Hamad Abdel Hadi, Muna Al Maslamani, Alaaeldin Abdelmajid

**Affiliations:** ^1^ Division of Infectious Diseases Communicable Diseases Centre Hamad Medical Corporation Doha Qatar; ^2^ Department of Clinical Pharmacy Communicable Diseases Center Hamad medical corporation Doha Qatar; ^3^ Department of Internal medicine Division of Family medicine Hamad medical corporation Doha Qatar; ^4^ 36977 Department of Internal medicine Division of Internal Medicine Hamad Medical Corporation Doha Qatar

**Keywords:** COVID‐19, cytokine storm, Filgrastim, interleukin 6, neutropenia, tocilizumab

## Abstract

Infection following SARS‐Co V‐2 leading to COVID‐19 disease is associated with significant morbidity and mortality. The clinical entity, COVID‐19 cytokine storm syndrome (CSS) is a severe immunological manifestation of the disease associated with ominous consequences. Tocilizumab is interleukin‐6 inhibitors that has been shown to hamper the catastrophic outcomes of CCS including the need for mechanical ventilation as well as reduce mortality, but the usage is limited by warnings of reactivation of potential latent infections or immune dysfunctions including severe neutropenia. We describe a case of 39‐year‐old Nepalese male patient with a background of scleritis maintained on azathioprine and rituximab therapy with normal baseline parameters including complete blood count who presented with acute COVID‐19 infection including associated leukopenia as well as severe neutropenia (absolute neutrophil count of 300 cells/µl), then progressed to critical disease culminating into CSS. Based on risks and benefits evaluation, the patient was treated with tocilizumab reinforced with granulocytes‐colony stimulating factor (G‐CSF, Filgrastim) to full recovery and safe outcome including reversal of neutropenia.

## Graphical abstract

For patients with COVID‐19 disease progressing toward cytokine storm syndrome (CSS) in the presence of significant neutropenia, combined tocilizumab and G‐CSF therapy circumvent potential therapeutic limitations leading to safe outcomes.

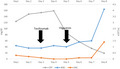

## 1 INTRODUCTION

SARS‐CoV‐2 virus is capable of causing severe respiratory tract infection culminating toward multiorgan dysfunctions and acute respiratory distress syndrome (ARDS),^1^ which is one of the commonest causes of prolonged mechanical ventilation leading to significant mortality.^2^ Although the full picture of the underlying mechanisms not well understood, but is frequently linked to the development of an intense hyperimmune response coined COVID‐19 cytokine storm syndrome (CSS), which characterized by simultaneous surge in immune activation leading to cascades of inflammatory response and subsequent organs failure where the cytokine interleukin‐6 (IL‐6) was found to play a pivotal role particularity, in early immune activation.^3^, ^4^ Tocilizumab is a recombinant monoclonal antibody of the IgG1 class, that binds to soluble and membrane‐bound IL‐6 receptors inhibit signaling pathways. It was licensed previously in the treatment of many rheumatological diseases.^5^ The efficacy of this drug to degrade immune activation including systemic inflammation in treating severe COVID‐19 patients was supported by evidence from randomized clinical trials in reducing associated mechanical ventilation and mortality.^6^

However, emergency licensing authorization warned of precautionary measure to limit usage in case of significant active infectious diseases such as tuberculosis, as well as to severe neutropenia with ANC <1000 cells /µl,^7^ which stems from concern of reactivations of infections or enhanced susceptibility for secondary opportunistic infections. These tangible risks have been highlighted in many observational studies being variable with the different biological agents.^8^ While balancing the effective benefits of Tocilizumab therapy during COVID‐19 disease specially CSS, its potential risks including precautions in patients with profound neutropenia must be carefully weighed. Moreover, such conditions have been highlighted as an indication to withheld or discontinue tocilizumab therapy. Nevertheless, experience during the pandemic circumvented many restrictions and precautions in favor of safe patients’ outcomes. Therefore, we outlay the clinical dilemma of using tocilizumab therapy in a patient with severe COVID‐19 and active CSS with potential fears of disease progression, in the context of profound clinical neutropenia. On risk benefits evaluation, the patient was treated with the effective therapy but covered with G‐CSF (Filgrastim) to safe outcome.

## 2 CASE REPORT

A 39‐year‐old male patient with a background history of probable autoimmune disease leading to undifferentiated scleritis was maintained by his ophthalmologist on azathioprine and rituximab therapy with stable disease for six years. The patient presented with typical respiratory and systemic symptoms of COVID‐19 disease including fever, dry cough, headache, and tiredness associated with nausea of one‐week duration.

Prescribed medications included azathioprine 150 mg daily and parenteral Rituximab 1000 mg injections last given 4 months prior to presentation. Prior to current presentation 3 months ago, complete blood counts including leukocytes parameter were within normal limits (WBC: 7.8 × 10^3^ µl, Hemoglobin: 13.4 gm/dL, Platelet: 437 × 10^3^/µl).

Initial evaluation demonstrated fever of 39.5°C, blood pressure of 103/64 mmHg, pulse rate of 95 per minute, respiratory rate of 22 per minute and oxygen saturation of 98% on room air with no signs of respiratory distress. Chest examination revealed evidence of bilateral basal crackles while the rest of the physical examination was unremarkable. Initial blood investigations showed: leucopenia with WBC count of 1.1 × 10 ^3^ µl, neutropenia with absolute neutrophils count (ANC) of 0.3 × 10^3^/µl, and lymphopenia of 0.6 × 10^3^µl, while peripheral blood smear revealed markedly decreased WBC with severe neutropenia and lymphopenia with reactive changes (Table 1). To assess for disease severity, severity biological markers were elevated: C‐reactive protein (CRP) 124 mg/L (Normal Value: less than 5 mg/L), ferritin 1185 µg/L (Normal value:30–490 µg/L), lactate dehydrogenase (LDH) 459 U/L (Normal Value: 135–225 U/L), and D‐dimer 4.18 mg/L (Normal Value: Less than 0.45 mg/L).

**TABLE 1:** White Blood Cell counts during patient's hospital stay

	Baseline (In January 2021)	Day 1	Day 2	Day 3	Day 4	Day 5	Day 6	Day 7	Day 8
WBCs (×103/µl)	7.8	1.1	0.9	0.9	1.1	1	1.4	1.5	4.1
ANC (×103/µl)	5	0.3	0.2	0.1	0	0	0.1	0.1	1.4
Lymphocytes (×103/µl)	2.1	0.6	0.6	0.7	0.8	0.7	1	1	1

Note

Day 3: Tocilizumab administered. Day 5: Filgrastim Administered.

**FIGURE 1:**
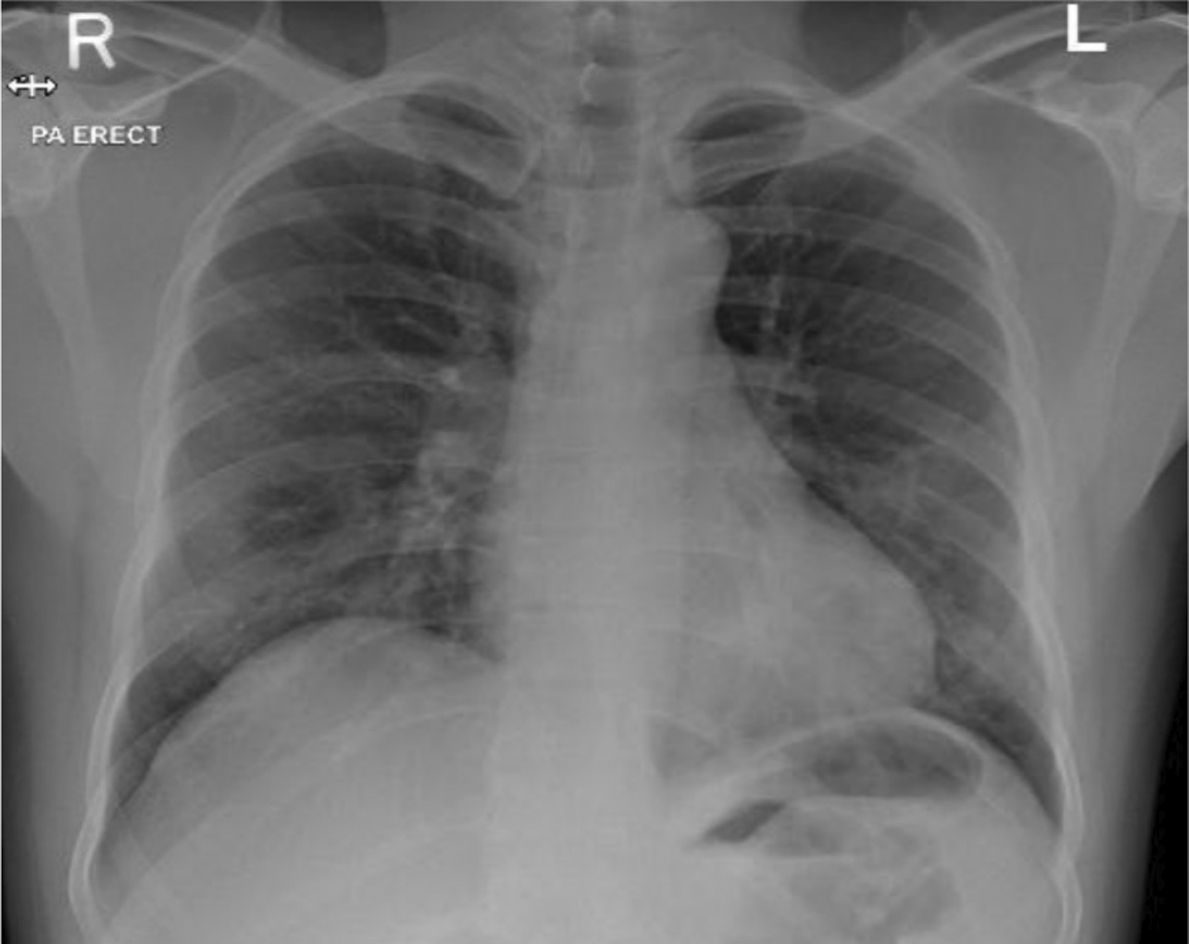
Initial Chest X‐ray upon presentation showed bilateral peripheral mid and lower lung zones patchy faint ground glass opacities

Chest X‐ray revealed bilateral infiltrates with visible hazy opacities (Figure 1). The clinical suspicion of moderate COVID‐19 disease was confirmed through COVID‐19 PCR with cycle threshold value (CT value) of 24 denoting early clinical disease.

The patient was admitted under airborne and contact precautions and started on the local protocol of favipiravir, ampicillin/sulbactam therapy together with venous thromboembolism prophylaxis in form of low molecular weight heparin in addition to symptomatic treatment.

**FIGURE 2:**
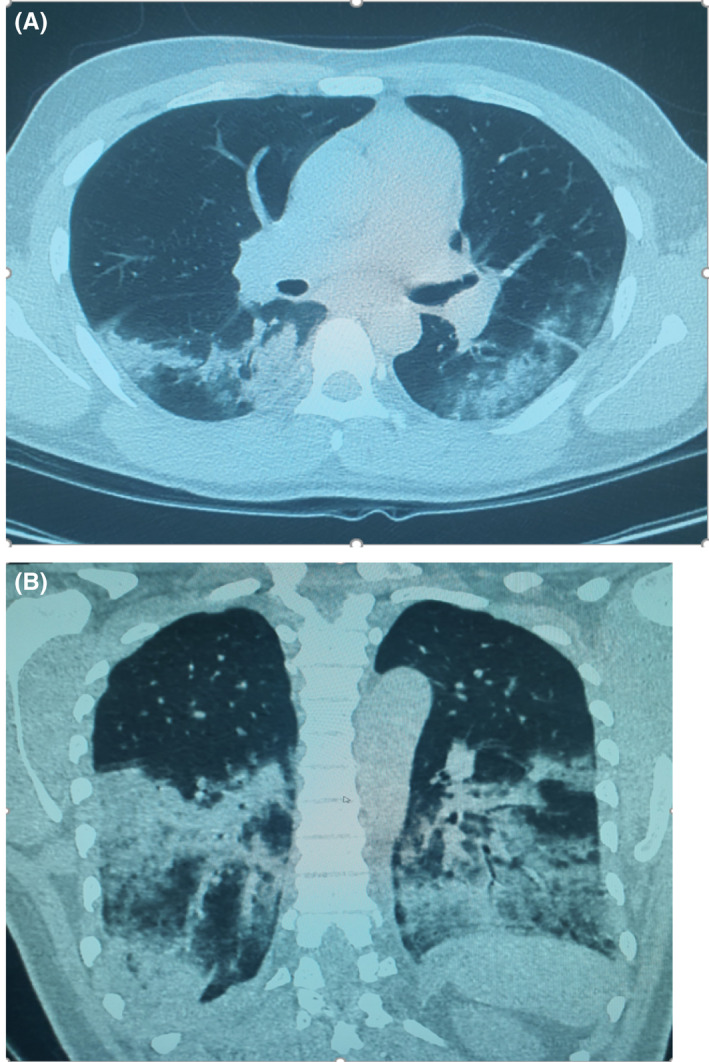
(A and B) CTPA study of the chest was performed to rule out pulmonary embolism at the time of deterioration of the patient condition, which was excluded by this study; however, the study reveals; Moderate degree of COVID‐19 pneumonia in the form of bilateral lower lung lobes extensive consolidations that are surrounded by ground glass opacities, as well as multi focal patches of ground glass opacities within the superior and inferior segments of the lingula of the left upper lung lobe. The right upper and middle lung lobes was spared

Because of the acute infection and fears of immune dysfunctions, prescribed immunosuppressive therapy was withheld. Over the following days, the patient condition deteriorated with high grade fever, worsening respiratory symptoms, and laboratory parameters including progressive neutropenia with a remarkable rise in D‐dimer to >35 mg/L as well as IL‐6 of 115 pg/mL (Normal Value: ≤7 pg/mL; Table 2). Searching for septic foci with repeated blood and urine cultures all returned negative. Associated pulmonary embolism was excluded at the time of clinical deterioration with CT pulmonary angiogram (CTPA) but confirmed bilateral infiltrative changes (with moderate CT COVID‐19 severity score; Figure 2). Despite these measures, the patient continued to deteriorate with respiratory compromise requiring higher oxygen supplementation. Escalation of management with broadening of the antibiotic coverage with piperacillin‐tazobactam, together with COVID‐19 specific therapy in form of 200 mg of parenteral Remdesivir therapy for the first day followed by daily 100 mg daily for the following 4 days as well as augmented management with 8 mg of parenteral dexamethasone therapy.

**TABLE 2:** Serial laboratory inflammatory markers during patient's hospital stay

	Day 1	Day 2	Day 3	Day 4	Day 5	Day 6	Day 7	Day 8
D‐Dimer (mg/L)	4.18	>35.2	>35.2		0.7	0.84	0.62	0.22
CRP (mg/L)	124.6	148	153.8	159.3	86.1	57.2	35.1	19.1
LDH (U/L)		459						
Ferritin (µg/L)	1185	6881				4776		
Procalcitonin (ng/ml)		3.86		2.28		0.81		0.16

Note

Day 3: Tocilizumab administered. Day 5: Filgrastim Administered.

**FIGURE 3:**
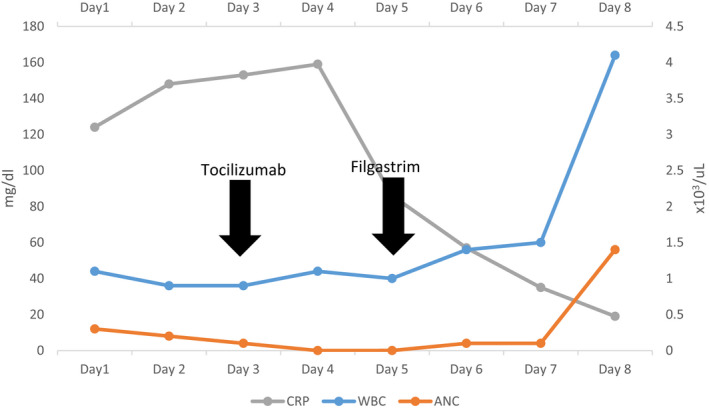
Patients serial WBC and CRP measurements during his hospital stay. ANC, absolute neutrophil count

The clinical assessment of an evolving COVID‐19 cytokine storm syndrome (CSS) was considered and the decisions of administering tocilizumab therapy was evaluated balanced between known benefits in pending CSS against the patient clinical condition of severe COVID‐19 infection with profound neutropenia. Eventually, 400 mg of parenteral tocilizumab was administered while closely monitoring clinical and laboratory parameters. Over the following days, there was gradual but remarkable improvement in clinical condition supported by falling of inflammatory parameters; however, the profound neutropenia was persistent. To curtail for that, the patient was started on G‐CSF in the form of Filgrastim 300 mcg twice daily with evident neutrophils rise within few days to ANC more than 1000 (Table 1; Figure 3).

During subsequent observation and hospital stay, the patient did not develop any active or opportunistic infections. Following recovery and consultation with managing ophthalmologist, the decision was made to continue withholding immune suppressive therapy pending progress and the patient was discharged to a safe outcome. Follow‐up revealed full recovery and no subsequent complications.

## 3 DISCUSSION

The spectrum of COVID‐19 disease manifest with variable clinical presentations ranging from asymptomatic to severe and critical disease encompassing the immunologically mediated condition cytokine storm syndrome (CSS) with significant morbidity and mortality. The clinical entity of CSS is characterized by an intense activation of the immune system with the release of various inflammatory mediators including cytokines such as interleukin 6 (IL‐6), IL‐10, and tumor necrosis factor α (TNF‐α) leading to systemic manifestations, end organs damage and eventually failure, and unfavorable outcomes.^9^

Because of circumstantial correlation with disease progression toward mechanical ventilation, prolonged hospital stays, significant morbidity and mortality, early detection of CSS is certainly beneficial. The evaluation is based upon multiple studied clinical and laboratory parameters which primarily heralded by new clinical deterioration in the cardiopulmonary status coupled with significant deterioration in laboratory parameters predominantly inflammatory markers such as WBCs, CRP, ferritin, LDH, and IL‐6.^10^ As a consequence, multiple guidance including the Infectious Diseases Society of America (IDSA) recommends the use of immune suppressive therapy such as tocilizumab for hospitalized adults with progressive severe or critical COVID‐19 disease who develops features of hyperinflammatory syndrome along with the standards of usual care such as steroids.^11^ Tocilizumab is a specific biological monoclonal antibody directed against anti‐IL‐6‐receptors blocking its signaling pathways to activate the immune system at cellular level. It was approved by FDA for various rheumatologic conditions and cytokine release syndrome associated with CAR‐T cell therapy.^12^

The therapeutic benefit of the drug was recognized early during the pandemic in hindering immune activation thus decreasing end‐organs dysfunctions and the need for intubation. Nevertheless, there were paucity of data about short‐term safety and tolerability of tocilizumab as in COVID‐19 disease. The majority of previous published data was mainly reported in the context of prolonged treatment of rheumatological conditions including rheumatoid arthritis. In a network metanalysis investigated potential adverse reactions in patients with any disease conditions except HIV, tocilizumab was associated with gastrointestinal disorders, dyslipidemia, elevated liver enzymes, and neutropenia. Moreover, the USA FDA’s black box warned against increased serious infections with most biological treatment such as active tuberculosis, bacterial infections, and invasive fungal infections.^13^ Therefore, the FDA emergency use authorization for tocilizumab in COVID‐19 disease; recommended screening and monitoring for existing active or latent infections as well as observing for associated neutropenia. Notably, the recommendations warned of cautious usage while monitoring in cases of remarkable neutropenia (ANC <1000 mm^3^) to the point of recommending against it to discontinue or withholding treatment if ANC is less than 300 mm^3^.^7^

Of note, leukopenia mainly driven by lymphopenia as well as thrombocytopenia is known manifestations of COVID‐19 disease since it was observed to correlate with disease severity and mortality.^14^

Furthermore, pan‐cytopenia including neutropenia has been reported as a feature of severe COVID‐19 particularly in the context CSS where bone marrow suppression or immune‐related cytopenia has been attributed as the underlying pathophysiological mechanisms.^15^

Conversely, although one of the adverse events of Azathioprine therapy is bone marrow suppression including neutropenia, our patient's blood parameters were maintained to normal levels while on treatment for six years. Usually, these established adverse events have a genetic predisposition since the recognized association stems from inadequate level of the degrading enzymes thiopurine methyltransferase (TPMT) and Nudix hydrolase (NUDT15) that metabolize azathioprine^16^

Nevertheless, our patient has stable blood counts for a long time before his current presentation, which indicates that his pan‐cytopenia is probably induced by infection. Taking that into consideration, our patient presented with grade 4 severe neutropenia (ANC 0.3 × 10^3^ µl) which most likely related to the severe acute COVID‐19 disease rather than prior immunosuppressants therapy with rituximab and azathioprine. The clinical dilemma encountered during management was of a patient with progressively severe COVID‐19 disease with established CSS who has concomitant profound neutropenia hindering admiration of Tocilizumab, an effective therapeutic agent that has been shown to alter disease progression and deterioration.

Despite the established management protocols and guidelines for severe COVID‐19 severe diseases among different patients’ categories, immunocompromised patients are often not included clinical trials making the clinical experience in immunocompromised hosts limited. However, published data showed that tocilizumab was used in treating severe disease in patients with utmost risk of immunosuppression including hematological malignancies and post Allogenic Hematopoietic Stem Cell Transplant with favorable and safe outcome.^17^, ^18^ Similarly, its utility in cancer patients (including those who are actively receiving chemotherapy) was reported and suggests its safe administration even among neutropenic patient's subset^19^, ^20^

In clinical practice, the golden ethical advice is to observe patient's autonomy while implementing the principles beneficence and doing no harm.^21^ Following discussion with the patients, balancing benefits against risks, the clinical team decided to administer Tocilizumab therapy under monitoring then salvage the critical neutropenia with G‐CSF (filgrastim) to stimulate neutrophil production to safe outcome and recovery. Although our approach was successful, the usage of G‐CSF in COVID‐19 disease is still controversial with some reports relates its administration to increase oxygen requirements, possible precipitation of cytokine storm and progression of alveolar damage.^22^, ^23^

This case tries to address two main issues: the use of tocilizumab in patients with severe COVID‐19 disease and profound neutropenia as well as G‐CSF therapy in the context of CSS and COVID‐19 disease. Since there are paucity of data and clinical experience to answer these questions during the evolving pandemic only accumulate evidence will participate toward building the needed experience. To the best of our knowledge, this will be the first case report to successful tocilizumab therapy in COVID‐19 patients with profound neutropenia. Further studies regarding tocilizumab benefits in such cohort of patients and whether the additive action of G‐CSF is recommended.

## 4 CONCLUSION

The COVID‐19 pandemic is evolving with significant morbidity and mortality especially from cytokine storm syndrome. Tocilizumab therapy is recommended to impede the deleterious consequences of CSS yet limited by precautionary measures in patients with severe neutropenia. In patients with severe COVID‐19 disease and critical neutropenia, we recommend the cautious administration of tocilizumab supported by G‐CSF on benefits against risks assessment.

## AUTHOR CONTRIBUTIONS

Ahmad Al Bishawi involved in final manuscript writing and editing. Shiema Abdalla and Marwa Askar involved in^:^initial draft writing. Wael Kanjo and Amal Sameer performed literature review. Gihan Mustafa involved in review and editing of the manuscript. Hamad Abdel Hadi involved in case supervision, literature review and final manuscript writing, editing and revision of the manuscript. Muna Al Maslamani and Alaaeldin Abdelmajid involved in supervision and final manuscript revision.

## CONFLICT OF INTEREST

The authors declare no conflict of interest.

## CONSENT

Written informed consent was obtained from the patient to publish this report in accordance with the journal's patient consent policy.

## Data Availability

The data that support the findings of this study are available from the corresponding author upon reasonable request.
